# Complete Genome of the Solvent-Tolerant *Staphylococcus warneri* Strain SG1

**DOI:** 10.1128/genomeA.00038-13

**Published:** 2013-03-14

**Authors:** Victor W. T. Cheng, Guijin Zhang, Kayode S. Oyedotun, Doug Ridgway, Michael J. Ellison, Joel H. Weiner

**Affiliations:** Department of Biochemistry, University of Alberta, Edmonton, Alberta, Canada

## Abstract

*Staphylococcus warneri* is a Gram-positive bacterium commonly found in human skin flora. The genome of a laboratory *S. warneri* isolate, strain SG1, was sequenced to explore its mechanism of solvent tolerance and its potential as a chassis for biofuel production.

## GENOME ANNOUNCEMENT

The adaptive mechanisms used to combat solvent stress are studied more rigorously in Gram-negative bacteria than in Gram-positive bacteria. This divergence occurs because the former bacterial group has an additional outer membrane compared to the latter group, and thus they are more tolerant to organic solvents in general. In recent years, the number of solvent-tolerant Gram-positive bacteria that have been isolated and studied has increased, especially those from the *Staphylococcus* and *Bacillus* genera. In this genome announcement, we present the complete genome of the solvent-tolerant strain *Staphylococcus warneri* SG1 that was isolated from our laboratory. The bacterium can tolerate up to 2.5% (vol/vol) butanol in rich liquid media, making it a suitable candidate chassis for biofuel production. Other strains of *S. warneri* have also been shown to be solvent tolerant (1, 2).

The *S. warneri* SG1 genome was sequenced at the University of Oklahoma Advanced Genome Centre using 454 FLX technology. *De novo* genome assembly was carried out independently by MIRA (3) and the CLC bio Genomics Workbench, followed by contig placement using the *Staphylococcus epidermidis* RP62A genome (accession no. NC_002976) (4) as a template. The SG1 genome, composed of the chromosome, a small plasmid, and at least one megaplasmid containing repetitive transposon sequences, is comprised of 2.56 Mb encompassing 2,429 predicted open reading frames (ORFs). Overall, the SG1 genome shares 99% and 95% identity with *S. warneri* strains VCU121 (accession no. AFEC00000000) and L37603 (accession no. NZ_ACPZ00000000), respectively, both of which have been deposited into GenBank as contigs. In addition to these two strains of *S. warneri*, the SG1 chromosome is also largely syntenous with those from *S. epidermidis* RP62A and *Staphylococcus aureus* COL. The 32.7% G+C content of the SG1 genome is consistent with values obtained from other *Staphylococcus* genomes. Sequences encoding hypothetical proteins and ORFs having only a general function prediction comprise 35.7% of the genome. A large prophage insertion showing similarities to common *S. aureus* prophage sequences (5) is also present on the chromosome. Genes conferring antibiotic resistance are located on transposable elements in the genome.

Gene ontology analyses using Kyoto Encyclopedia of Genes and Genomes (KEGG) and Clusters of Orthologous Groups (COG) designations of protein function revealed distinctions between *S. warneri* and *S. epidermidis/aureus*. The following proteins were lacking in the latter species but present in SG1: a restriction-modification endonuclease system; an *S*-adenosyl-l-methionine-dependent methyltransferase (AGC89472.1); a fusion protein comprising an N-terminal phosphatase and a C-terminal VanW domain that is implicated in vancomycin resistance (6); enzymes to convert chorismate to 4-aminobenzoate, a folic acid precursor; cell wall and peptidoglycan modification enzymes; glucuronate interconversion enzymes; and various proteases, transcriptional factors, enzymes, and hypothetical proteins. Interestingly, COG functional annotation of AGC89472.1 putatively assigned it to be a cyclopropane fatty acid synthase, which plays an important role in the adaptation to solvent stress in *Pseudomonas putida* and *Oenococcus oeni* (7, 8). An *N*-acetylmuramoyl-l-alanine amidase (AGC90872.1) involved in peptidoglycan synthesis might be linked to the increased lysostaphin resistance shown by SG1 cells upon exposure to an organic solvent.

### Nucleotide sequence accession numbers.

The nucleotide sequences have been deposited into GenBank under the accession no. CP003668 to CP003676.
